# Characterising Dissolution Dynamics of Engineered Nanomaterials: Advances in Analytical Techniques and Safety‐by‐Design

**DOI:** 10.1002/smll.202500622

**Published:** 2025-06-02

**Authors:** Swaroop Chakraborty, Eugenia Valsami‐Jones, Superb K. Misra

**Affiliations:** ^1^ School of Geography Earth and Environmental Sciences University of Birmingham Birmingham B152TT UK; ^2^ Materials Engineering Indian Institute of Technology Gandhinagar Gujarat 382355 India

**Keywords:** dissolution, nanomaterials toxicity, nanomaterials transformations, nanoparticle solubility, safe‐by‐design

## Abstract

Engineered Nanomaterials (ENM) have rapidly emerged as vital components in modern technology, most notably as vehicles in vaccine delivery, which highlights their growing potential for interaction with biological and environmental systems. One critical property influencing ENM behavior is dissolution, the release of ions and molecules into surrounding media, which dictates their abundance, fate, and biological response. A decade ago, dissolution was recognised as pivotal in understanding ENM interactions with exposure media and assessing their potential toxicity. Since then, progress in this field has led to a deeper understanding of ENM surface chemistry and transformations, positioning dissolution as a key factor in achieving “Safety‐by‐Design” (SbD) for sustainable ENM applications. Early dissolution studies relied on batch and flow‐through methods, such as dialysis, but recent advances have favored in situ techniques such as single‐cell/single‐particle inductively coupled plasma mass spectrometry (ICP‐MS) and liquid‐cell electron microscopy, enabling real‐time dissolution measurements. Additionally, computational models can now predict ENM reactivity and stability, enhancing the understanding of dissolution behavior. This perspective critically examines these developments, highlighting computational approaches for their efficiency and scalability, and proposes a roadmap to integrate these insights with SbD goals for safer, sustainable nanotechnology applications.

## Graphical abstract

The perspective highlights how advanced analytical and computational tools—from batch tests and isotope labeling to in situ Electron Microscopy and Artificial IntelligenceI/Machine Learning—enable a deeper understanding of Engineered nanomaterial dissolution. These insights are crucial for controlling, regulating, and leveraging dissolution dynamics to guide Safety‐by‐Design strategies for sustainable nanotechnology.

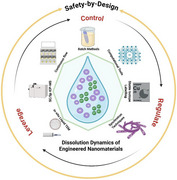

## 1 Dissolution: An Integral Part of Engineered Nanomaterial Characterisation

Since it was first observed that materials at the nanoscale exhibit distinct properties compared to their bulk counterparts, engineered nanomaterials (ENM) have become integral to advancements across multiple domains of applications.^[^ ^1^ ^]^ The diverse range of properties offered by ENM—dependent on attributes such as shape, size, and surface chemistry—allows for precise tuning of thermal, redox, optical, and magnetic behaviors.^[^ ^2^ ^]^ A combination of intrinsic material properties and extrinsic factors, including exposure conditions, can lead to complex interactions within biological and environmental systems.^[^ ^3^, ^4^ ^]^ This has necessitated the integration of “safety‐by‐design” (SbD) framework, aiming to mitigate risks across the ENM lifecycle from initial design to end‐of‐life disposal.^[^ ^3^, ^5^ ^]^ SbD framework integrate material design with strategies to minimise risks and enhance functionality by predicting and controlling ENM behavior throughout their lifecycle. This approach fosters safer applications in technology and the environment while ensuring sustainability.^[^ ^6^ ^]^ ENM are predominantly synthetic, metastable structures that exist far from thermodynamic equilibrium, meaning they possess elevated free energies compared to bulk phases. According to Vogelsberger (2003), such nanomaterials can be viewed on a free energy “landscape,” where the system's total free energy depends on both particle size and concentration.^[^ ^7^ ^]^ Under environmental or biological conditions, the fundamental driving force behind ENM dissolution is the thermodynamic propensity to relax toward more stable, equilibrium forms through spontaneous reactions with surrounding constituents (e.g., oxygen, water, or redox‐active biomolecules). This relaxation process, effectively lowering the Gibbs free energy, explains why zero‐valent metals like iron oxidize to iron oxide upon exposure, or why other reactive nanoparticles (e.g., Cu, Ag) dissolve in oxidative or mildly acidic environments.^[^ ^8^, ^9^ ^]^ For instance, metallic iron nanoparticles rapidly oxidise to iron oxide species upon environmental exposure, driven by the highly negative Gibbs free energy associated with iron oxidation. In this framework, particle‐specific properties—such as size, shape, or surface functionalisation, act primarily as kinetic modulators, altering the rate at which a nanoparticle moves along the free energy landscape rather than changing the fundamental thermodynamic endpoint. Smaller particles, for instance, tend to dissolve more rapidly because they feature higher specific surface area and surface energy, accelerating movement toward a lower‐energy state. However, the thermodynamic remains largely affected by the chemical composition and its reaction‐free energy with the medium, not merely morphological details. Consequently, clearly distinguishing intrinsic thermodynamic instability (i.e., elevated Gibbs free energy) from secondary kinetic effects (i.e., how quickly dissolution proceeds) is crucial. This distinction helps avoid confusion, especially for new researchers in the field—by highlighting that, while particle size or shape may influence dissolution rates, the primary driver is the free energy reduction that arises when metastable ENM form or dissolve into more stable phases.^[^ ^8^, ^9^ ^]^ The dissolution of ENM holds substantial environmental and biological significance. Dissolution directly affects the bioavailability of ENM, influencing critical biological interactions such as cellular internalization, oxidative stress, enzymatic perturbations,^[^ ^10^ ^]^ and resultant toxicity in various organisms. Environmentally, dissolution determines the persistence, mobility, chemical reactivity, and transformation pathways of ENM, ultimately influencing their fate and interaction with ecosystem components. For example, silver nanoparticles (Ag NPs) with higher intrinsic solubility may release ions that are more readily excreted or transformed, limiting their accumulation.^[^ ^11^, ^12^ ^]^ By contrast, lower‐solubility ENM—such as certain TiO₂—can persist longer if not effectively broken down, potentially altering exposure and risk profiles. This highlights how solubility fundamentally shapes the fate and impact of ENM in real‐world settings. Consequently, a clear understanding of dissolution behavior is pivotal for accurately evaluating ecological risks, guiding regulatory decisions, and informing sustainable and SbD nanotechnologies.

Over the past decade, significant advancements in understanding ENM dissolution have highlighted that ion release rates are influenced not only by ENM properties (e.g., shape, size, surface chemistry, and roughness) but also by external factors such as media composition, pH, ionic strength, and the presence of organic ligands^[^ ^13^, ^14^, ^15^ ^]^ Among the various factors influencing ENM dissolution, the chemical composition and intrinsic thermodynamic properties of ENM are fundamentally the most critical determinants. ENM are primarily chemical substances, each possessing unique equilibrium solubility and chemical reactivity profiles determined by their intrinsic thermodynamic stability. This inherent chemical identity governs their fundamental tendency to interact with environmental or biological media. For example, metallic ENM such as metallic iron (Fe), copper (Cu), or silver (Ag) exhibit dissolution behavior primarily driven by the inherent chemical reactivity and oxidation potentials, independent of particle size or shape.^[^ ^16^ ^]^ For instance, Ag NPs are inherently prone to dissolution in aqueous environments due to their relatively low reduction potential and strong affinity for sulfur‐containing biomolecules, thereby readily releasing Ag⁺ ions under physiological and environmental conditions.^[^ ^17^ ^]^ Conversely, titanium dioxide (TiO₂) nanoparticles exhibit much lower dissolution owing to their high thermodynamic stability and low aqueous solubility, primarily releasing ions under strongly acidic or photochemically activated conditions.^[^ ^18^ ^]^ Similarly, zinc oxide (ZnO) nanoparticles rapidly dissolve under mildly acidic biological or environmental conditions due to their intrinsically higher solubility and lower thermodynamic stability compared to more inert oxides such as SiO₂ or CeO₂ nanoparticles.^[^ ^19^ ^]^ These examples highlight that intrinsic chemical composition sets the baseline for nanoparticle dissolution, whereas particle‐specific characteristics such as size, shape, or surface chemistry modulate dissolution kinetics without overriding these fundamental chemical tendencies. Thus, when designing ENM with SbD framework, prioritising chemical composition and the intrinsic stability profile is critical. Subsequent tuning of secondary factors (e.g., size, surface coatings) then enables fine control of dissolution rates and environmental or biological interactions.

**Figure 1:**
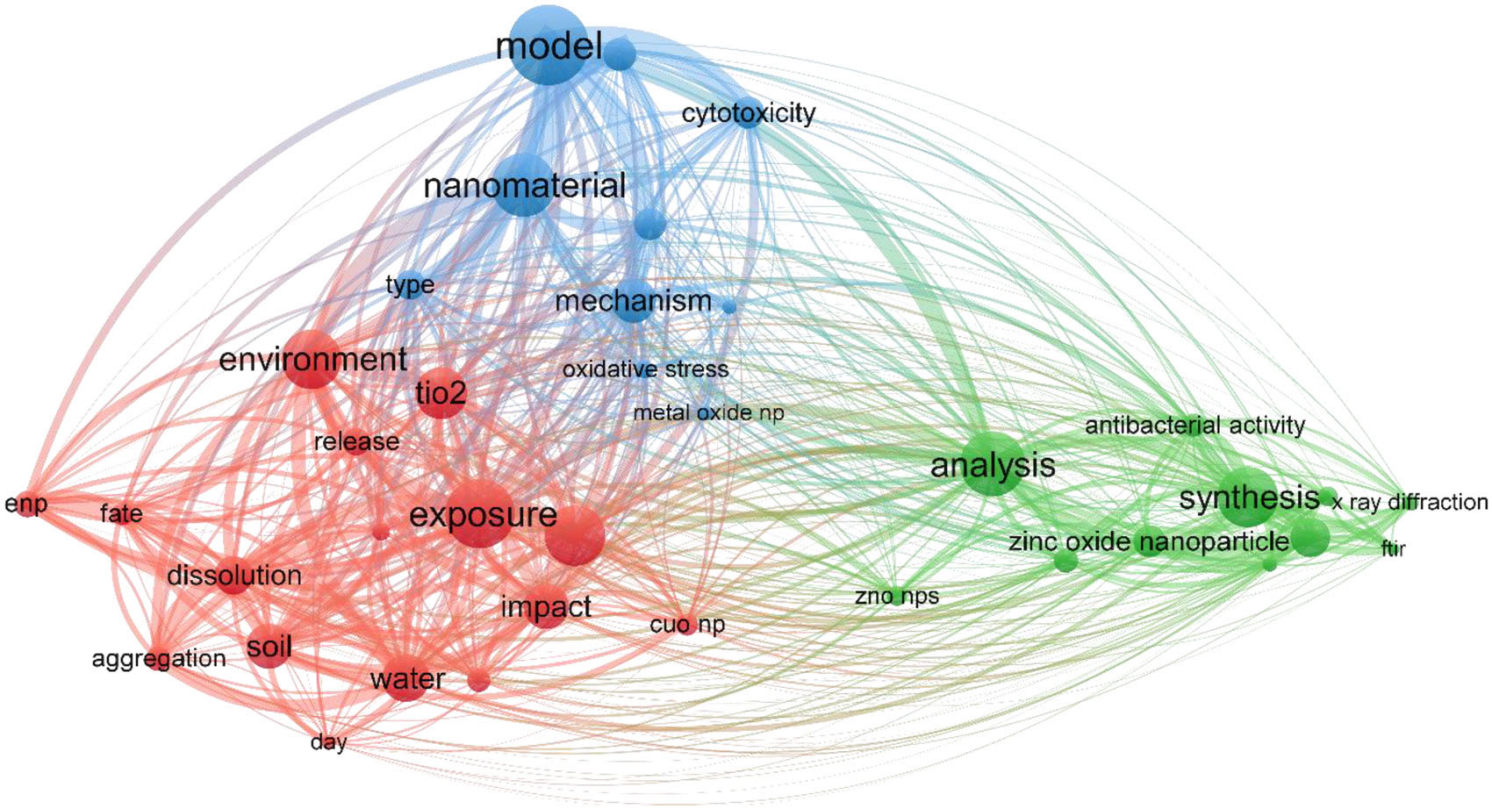
Keyword co‐occurrence network illustrating the multidisciplinary landscape of nanomaterial dissolution research. This network, generated using VOSviewer, highlights the interconnectedness of key terms relevant to dissolution, exposure, and environmental impact of ENM. Clusters are color‐coded to represent major themes, such as environmental behavior and fate (red), mechanistic insights and modeling (blue), and synthesis and analysis of nanomaterials (green). Prominent terms like “dissolution,” “exposure,” and “mechanism” highlight the importance of understanding nanomaterial transformations and their implications for toxicity and environmental safety. This figure demonstrates the broad scope of research addressing dissolution dynamics and their role in advancing SbD approaches. A total number of 40 keywords with a minimum threshold of occurrence of 70 were considered for this web chart. A total number of 1000 research papers were considered from years 2015 to 2025.

Dissolution, the process by which ENM release ions when exposed to biological or environmental fluids, is now recognised as a critical parameter in SbD framework due to its central role in determining ENM behavior in ecosystems.^[^ ^20^ ^]^ Dissolution encompasses both equilibrium solubility (a thermodynamic measure of the material's potential to dissolve) and dissolution rate (a kinetic measure that reflects how rapidly the process occurs).^[^ ^21^ ^]^ Together, these parameters influence the potential of ENM to release ions, impacting cellular uptake mechanisms and their interactions with organisms and ecosystems. The relevance of dissolution extends beyond ion release, influencing the material's transformation, persistence, and overall fate in biological and environmental systems.^[^ ^22^, ^23^, ^24^ ^]^ Furthermore, research has demonstrated that dissolution behaviors can be highly variable across different ENM and applications, underscoring the importance of tailoring SbD strategies to the specific properties of each material.^[^ ^13^, ^25^ ^]^ SbD is a paramount concept in the context of ENM, focusing on the development of safe and functional materials and nano‐enabled products.^[^ ^26^ ^]^ The SbD approach is transformative, moving beyond merely identifying hazards associated with inherently unsafe nanomaterials. It fosters a comprehensive understanding of the properties that make ENM safe or potentially hazardous.^[^ ^25^ ^]^ This strategic approach not only enhances safety but also facilitates innovation in industrial processes in a structured manner. The interconnected nature of research into ENM dissolution is exemplified by the keyword co‐occurrence network shown in **Figure** **1**. The network reveals distinct thematic clusters, including dissolution and environmental interactions, mechanistic insights and modeling approaches, and synthesis and material characterisation. The prominence of terms such as “dissolution,” “exposure,” and “environment” reinforces the critical role of understanding ENM transformations in evaluating their safety and environmental impact. This multidisciplinary focus aligns with the paper's objective to provide a comprehensive perspective on dissolution as a central parameter in SbD strategies.

The dynamic and complex nature of ENM dissolution has driven the development of an array of sophisticated analytical techniques over the last decade. Established methods, such as dialysis and stable‐isotope labeling, have been complemented by advanced in situ characterisation tools like liquid‐cell electron microscopy and single‐cell/single‐particle inductively coupled plasma mass spectrometICP‐MS), allowing for observation of dissolution processes with high precision in biologically relevant environments.^[^ ^22^, ^27^, ^28^, ^29^ ^]^ These advancements reflect a concerted effort to capture dissolution dynamics with greater accuracy and in increasingly realistic settings, aligning closely with SbD framework aimed at evaluating ENM safety in relevant biological or environmental contexts. Despite these technological strides, one of the major challenges in the field remains the standardisation of dissolution assessment methods. The diversity of ENM properties and application contexts makes it challenging to create a unified framework for dissolution testing that can account for both intrinsic material characteristics and relevant environmental conditions. Recent European projects like SUNSHINE, DIAGONAL, and CompSafeNano^[^ ^6^ ^]^ have recognised this gap, aiming to develop dissolution metrics that support SbD and contribute to regulatory frameworks, with the goal of achieving broader adoption of SbD framework in nanotechnology.

As the field of nanotechnology continues to evolve, understanding dissolution dynamics is increasingly recognised as pivotal to achieving SbD and broader sustainability objectives. The role of dissolution as a predictor of ENM behavior in biological and environmental systems highlights its potential as a benchmark for safe design practices. Integrating dissolution into SbD framework offers a pathway to minimise risks associated with ENM use, reduce environmental impacts, and align material design with sustainability goals. Despite considerable advances in the understanding of dissolution dynamics over the past decade, recent literature lacks a cohesive perspective encompassing the evolution from classical batch approaches to state‐of‐the‐art real‐time analytical techniques, particularly with an emphasis on SbD. Through this perspective, we discuss ways to integrate the latest experimental and computational developments. As iterated earlier, sp/sc‐ICP‐MS and liquid‐cell electron microscopy provide invaluable real‐time insights into ENM transformation; building on these, emerging computational approaches, particularly machine learning, are now enabling efficient, large‐scale prediction of ENM dissolution behaviour. By providing a comprehensive overview of these interdisciplinary advancements, this perspective is positioned to guide future research directions and regulatory frameworks, establishing dissolution as a central metric for safer and sustainable nanotechnology. While this perspective does not aim to catalog dissolution behaviors across all ENM, readers seeking comprehensive overviews of material‐specific dissolution profiles are referred to recent reviews that detail solubility, dissolution kinetics, and media‐specific transformations across a broad spectrum of ENM.^[^ ^19^, ^30^, ^31^, ^32^, ^33^, ^34^ ^]^ These resources complement our focus by offering quantitative comparisons and solubility trends, whereas this paper highlights emerging tools and approaches for characterising dissolution and advancing SbD strategies.

## 2 Evolving Understanding of ENM Dissolution

**Figure 2:**
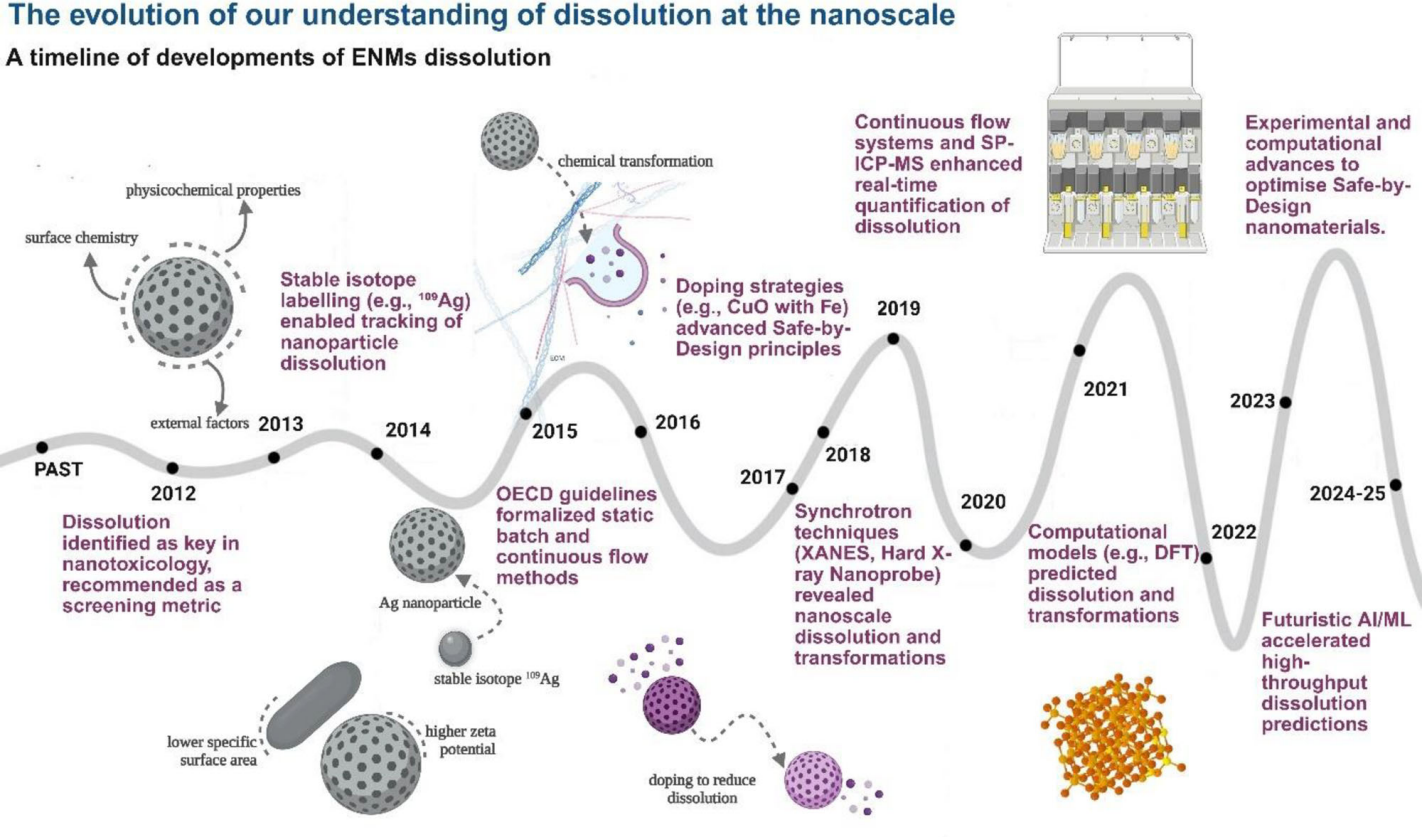
The evolution of our understanding of dissolution at the nanoscale. This timeline illustrates significant advancements in ENM dissolution research, beginning with the identification of dissolution as a key parameter in nanotoxicology post‐2010. Breakthroughs include stable isotope labeling in 2013 for nanoparticle tracking, formalisation of OECD guidelines in 2015, and SbD strategies like doping to control dissolution in 2016. Subsequent years saw the rise of continuous flow systems and sp‐ICP‐MS for real‐time dissolution measurements, synchrotron techniques for nanoscale insights, and computational models for predictive analytics. Post 2023, AI/ML‐based high‐throughput prediction systems emerged, promising to accelerate dissolution research and optimise SbD frameworks for nanotechnology (Figure made using Biorender software).

Traditionally, the concept of dissolution has been rooted in disciplines like analytical chemistry, geochemistry, and physical chemistry. Foundational theories such as the Noyes‐Whitney, Gibbs‐Thomson, and Ostwald‐Freundlich equations, along with concepts like thermodynamic driving forces, critical nucleus size, and Ostwald ripening, have been instrumental in shaping our understanding of solubility and dissolution processes.^[^ ^35^, ^36^ ^]^ However, applying this foundational knowledge to nanoscale systems introduces challenges, particularly in regulatory contexts, as the high surface area‐to‐volume ratio of ENM necessitates consideration of surface and bulk properties in tandem, unlike traditional bulk materials.^[^ ^37^ ^]^ **Figure** **2** shows the way the understanding of ENM dissolution evolved with time, highlighting key advancements and classical papers reporting pivotal breakthroughs in dissolution mechanisms, analytical techniques, and SbD strategies for nanomaterials. A growing body of work has focused explicitly on comparative chemical kinetics, redox potentials, and alloying strategies as key determinants of nanoparticle dissolution. For instance, Adamczyk et al.^[^ ^38^ ^]^ presented a theoretical framework for the oxidative dissolution of Ag NPs, clarifying how redox potentials drive both dissolution rates and morphological evolution under oxidative stress. In parallel, Chang et al.^[^ ^39^ ^]^ highlighted how surface oxidation in layered 2D materials can be systematically mitigated by controlling thermodynamic driving forces in device applications, thus shifting the dissolution (or oxidation) pathway. Wang et al.^[^ ^40^ ^]^ similarly demonstrated, in the context of emerging 2D nanomaterials (e.g., MoS₂, graphene derivatives), that the intrinsic chemistry and redox reactivity dictate a material's dissolution profile, impacting downstream biological interactions and environmental fate. Finally, Cipriano et al.^[^ ^41^ ^]^ address how tuning the chemical composition of binary alloy nanoparticles—through controlled alloying and doping—can markedly reduce dissolution by adjusting their intrinsic thermodynamic instability. These examples reaffirm that while size, shape, and surface morphology modulate dissolution kinetics, the most decisive factor is the nanoparticle's chemical identity, especially in oxidative or reductive environments where Gibbs free energy changes dominate. Moreover, the potential to engineer ENM by tweaking redox potentials or their compositions offers a powerful means for SbD, ensuring that nanoparticles maintain structural integrity or predictable dissolution rates in diverse real‐world scenarios.

In the context of Environmental Health and Safety, the role of dissolution first gained attention for its utility in toxicological interpretations.^[^ ^19^, ^42^ ^]^ Initially treated as a chemical phenomenon, dissolution was later integrated into the broader concept of “bio‐durability” when ENM were observed to translocate across biological barriers.^[^ ^29^, ^42^ ^]^ The potential for dissolution‐driven transformations and the release of bioavailable ions introduced new dimensions in assessing ENM toxicity, particularly as toxicological studies began correlating ENM physicochemical properties with biological responses for applications like antimicrobial and anticancer therapies.^[^ ^43^ ^]^ Early work focused heavily on particle internalisation and its effects, predominantly studied via microscopy,^[^ ^44^ ^]^ but as the link between ENM dissolution and biological response was increasingly established, precise ion release characterisation became a crucial parameter.^[^ ^45^ ^]^

The dissolution process of ENM is significantly influenced by the specific environmental conditions of the surrounding solution, particularly factors such as pH, ionic strength, and the presence of natural organic matter (NOM).^[^ ^46^ ^]^ pH profoundly affects dissolution by altering nanoparticle surface charge and influencing ion speciation in solution; typically, acidic conditions accelerate dissolution due to increased protonation and enhanced solubility of metal‐based nanoparticles, while neutral or basic conditions tend to reduce dissolution rates or favor reprecipitation of secondary phases.^[^ ^19^, ^47^ ^]^ NOM, ubiquitous in environmental matrices, interacts with ENM surfaces by forming surface coatings or complexes, which have been shown to influence dissolution. For example, NOM can stabilise nanoparticles by forming a protective layer around the surface, significantly reducing ion release rates; conversely, certain NOM fractions can chelate metal ions, driving dissolution equilibria forward and facilitating greater nanoparticle dissolution.^[^ ^13^ ^]^ Additionally, ionic strength and the presence of specific ions (e.g., chlorides, sulfates, or phosphates) strongly affects ENM dissolution through competitive adsorption, ion‐pair formation, or precipitation of sparingly soluble mineral phases on nanoparticle surfaces.^[^ ^48^ ^]^ Increased ionic strength generally compresses the electrical double layer around nanoparticles, enhancing aggregation and subsequently reducing surface exposure and dissolution.^[^ ^22^, ^49^ ^]^ Understanding these nuanced environmental interactions is critical, as they determine the actual dissolution behavior and bioavailability of ENM under realistic exposure scenarios, thereby informing accurate risk assessment and effective SbD approaches. Post‐2010, dissolution was largely identified as essential for understanding how compositional variations in ENM influence cellular responses.^[^ ^19^ ^]^ This recognition positioned dissolution as a key factor in toxicity screening, prompting recommendations for adapting and developing techniques for dissolution characterisation in biological media. Although dissolution mechanisms at the nanoscale were not fully understood then, post‐2010 literature highlighted an uptick in studies linking dissolution data with toxicological outcomes.^[^ ^50^ ^]^ Research papers emerging post‐2010 started showing shape and surface features of ENM could be engineered to modulate dissolution, sparking interest in SbD strategies. By 2015,^[^ ^50^ ^]^ SbD approaches began incorporating strategies like doping to control dissolution rates, exemplified by work with CeO₂, SiO₂, CuO, and Ag NPs, where controlled dissolution aligned with safer design principles. As dissolution was increasingly recognissed as a predictive marker of ENM behavior, the field saw debates around “sparingly” soluble ENM, whose dissolution patterns challenged the binary classification of ENMs as either highly soluble or insoluble.^[^ ^20^, ^51^ ^]^ Researchers recommended leveraging nano‐informatics to synthesie environmental health requirements, accommodating a wider range of dissolution behaviors.^[^ ^52^, ^53^ ^]^ The complexities in assessing ENM dissolution tend to have cumulative effects on dissolution kinetics. Major challenges in assessing dissolution at the nanoscale are a) the lack of universal processes for separating ENM from their dissolved species in complex media, b) measuring dissolution in complex backgrounds and matrices, and c) the lack of in situ techniques to measure dissolution in the macroscopic environment. The separation of the nano‐particulate form from the dissolved component is a pivotal aspect; an ineffective method of separation can lead to over/under‐representation of dissolution.

**Figure 3:**
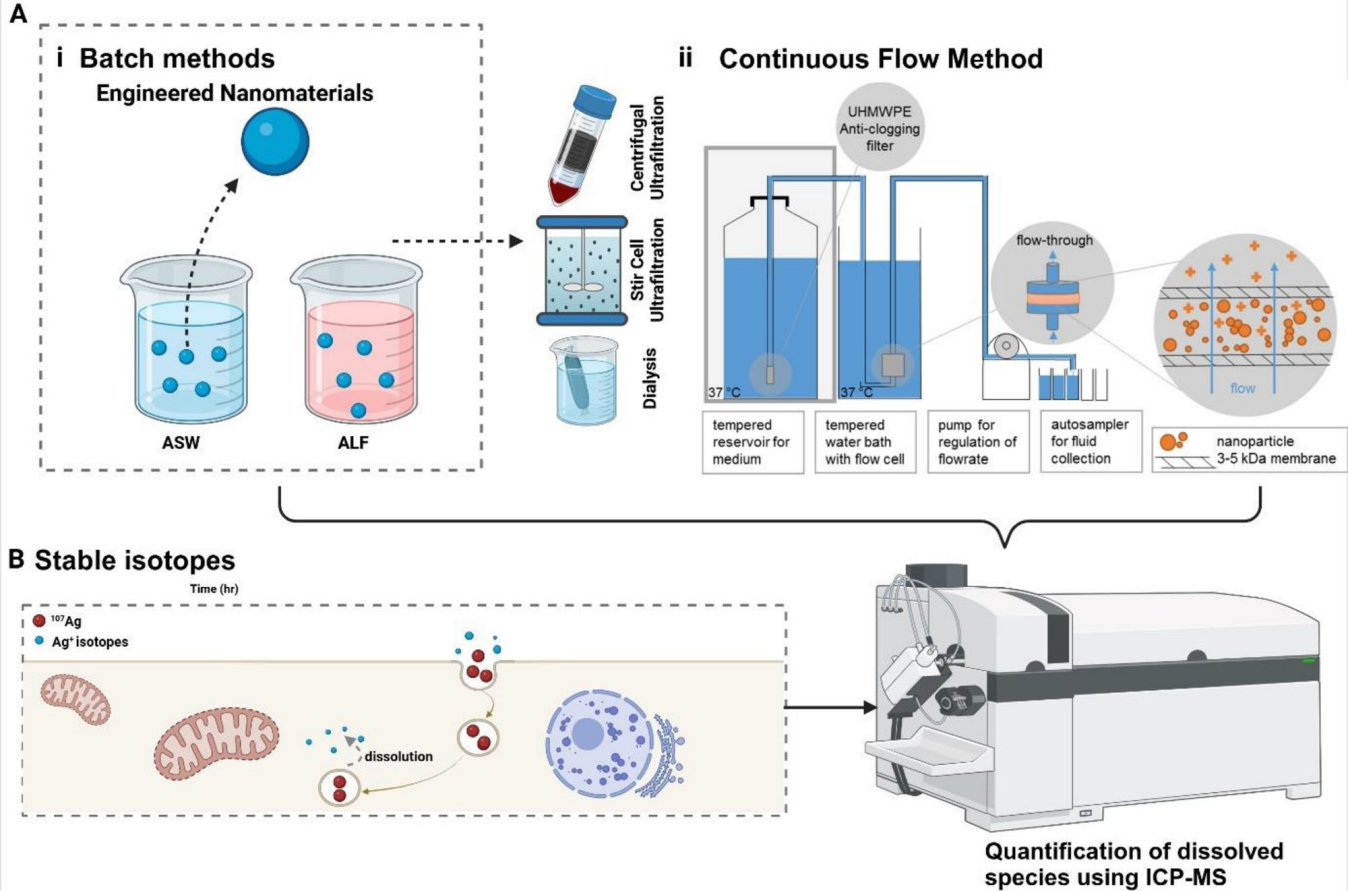
Methods for assessing dissolution behavior of ENM. (A) Experimental approaches for studying dissolution: (i) Batch Methods: Engineered nanomaterials are suspended in artificial seawater (ASW) or artificial lysosomal fluid (ALF) to simulate environmental or biological conditions, respectively. Techniques such as dialysis, stir‐cell ultrafiltration, and centrifugal ultrafiltration are employed to separate dissolved substances from nanoparticles. (ii) Continuous Flow Method: A sophisticated setup incorporating a tempered reservoir and water bath ensures controlled conditions, while an ultrahigh molecular weight polyethylene (UHMWPE) anti‐clogging filter and 3–5 kDa ultrafiltration membrane allow separation of nanoparticles and their dissolved ions. Autosamplers enable continuous sampling of fluid for precise dissolution measurements. Figure adapted with copyright permission from.^[^ ^59^ ^]^ (B) Stable Isotope Tracing: The use of isotopically labeled nanoparticles (e.g., ^107^Ag) allows for high‐resolution tracking of dissolution over time, distinguishing between particulate and dissolved species. Dissolution processes can be monitored in biological systems and quantified using advanced analytical techniques like ICP‐MS. This figure highlights the integration of batch, continuous flow, and isotope‐labeling methods to achieve comprehensive and precise measurements of nanomaterial dissolution. (Figure made using Biorender software).

Dissolution assessment of ENM is now being standardised by the Organisation for Economic Co‐operation and Development (OECD) through two methods (i.e., static batch dissolution and continuous flow dynamic dissolution).^[^ ^54^, ^55^ ^]^ While most protocols for macroscopic materials dissolution can be adapted to study the process at the nanoscale, most of the challenges encountered are associated with complexities arising from the vast landscape of physicochemical properties. The static batch method (**Figure** **3**A‐i) is suitable for equilibrium studies, where ENM are incubated in a controlled medium for a specified time, followed by the separation of dissolved species from particulates using techniques like filtration, centrifugation, or dialysis. This approach is effective for evaluating dissolution behavior under static conditions, such as in environmental or biological media. Conversely, the continuous flow dynamic dissolution (Figure 3A‐ii) method is designed to replicate non‐equilibrium, real‐time dissolution kinetics by subjecting ENM to a continuous flow of the testing medium, mimicking dynamic systems such as blood flow or aquatic environments. This method allows for the continuous collection and analysis of dissolved fractions using high‐resolution techniques like ICP‐MS or AAS. The OECD emphasises the importance of accurate separation techniques, appropriate media selection reflective of realistic exposure scenarios (e.g., artificial lysosomal fluid or seawater), and complementary analytical methods to ensure robust and reliable data. These methods provided a standardised framework for understanding ENM dissolution behavior, enabling risk assessment and supporting SbD framework.

Dialysis is widely recognised as a viable technique for quantifying the dissolution of ENM but only aligns partially with OECD recommendations. This method offers advantages such as ease of sample collection, low sampling volumes, and reduced experimental costs.^[^ ^22^ ^]^ However, its effectiveness hinges on the diffusion process through membranes, which is inherently slow, potentially leading to inaccurate interpretations of dissolution kinetics.^[^ ^56^ ^]^ Challenges with ultracentrifugation mirror those of dialysis, including prolonged separation times, often exceeding 30 minutes. The effectiveness of separation is influenced by the physical forces applied, pore size limitations, and interactions between the ENM and the separation medium. Optimising separation techniques is critical for ensuring accurate recovery of dissolved species, especially in complex media. Such optimisation is necessary across different nanoparticle systems to provide reliable dissolution data that underpin regulatory compliance and SbD strategies. OECD guidelines suggest that dialysis is not recommended for separating ENM and their dissolved substances due to its slow diffusion process, which may lag behind the dissolution rate, and the potential for sample loss or alteration during handling. In contrast, centrifugal ultrafiltration is preferred as it efficiently separates ENM from dissolved substances. This technique uses centrifugal force to drive the sample mixture through an ultrafiltration membrane, allowing dissolved substances to pass through while retaining ENMs. The filtered media containing dissolved substances can then be analysed, making it a more reliable method for dissolution studies compared to dialysis.^[^ ^55^ ^]^

## 3 Advanced Analytical Techniques for Measuring Dissolution

In principle, ENM dissolution can be measured both qualitatively and quantitatively. For qualitative measurements, the separation of particulates from dissolved species is not essential. Analytical techniques such as dynamic light scattering (DLS), ion selective electrodes, and electron microscopy have been extensively used to assess dissolution and substantiated with mass spectrometry techniques such as ICP‐MS/OES. In 2010, Li et al^[^ ^57^ ^]^ explored how dissolution influences the aggregation behavior of Ag NPs under varying environmental conditions. Using DLS, the authors monitored changes in hydrodynamic size, revealing dissolution‐driven size reductions from ≈60 to 50 nm, alongside aggregation‐induced increases up to 100–150 nm, depending on ionic strength and stabilizing agents. Higher ionic strength was found to accelerate dissolution and promote aggregation due to silver ion release, which destabilised the nanoparticles. This research, highlights the complex interplay of dissolution and aggregation, emphasising their significance in environmental and toxicological assessments of Ag NPs. The reduction in the hydrodynamic size of ENM with time in dissolving media, such as artificial lysosomal fluid (ALF), is inferred as a measure of dissolution. For instance, when 50 µg mL^−1^ of CuO NPs were suspended in ALF, the hydrodynamic size reduced from 165 to 15 nm.^[^ ^22^ ^]^​ The DLS data was corroborated with dissolution measurements using atomic absorption spectrometry (AAS). These results, however, could be misleading in other cases. ENM are suspended in media with high ionic strength (such as artificial sea water (ASW)), agglomerate in the medium, and sediment to the bottom of the DLS cuvettes. In such cases, hydrodynamic size shows a similar trend, due to the sedimentation of the majority of NPs. For example, Chakraborty et al (2018) reported a reduction in the hydrodynamic size of 50 µg mL^−1^ of CuO NPs when suspended in ASW, from 470 nm to 210 nm after 10 h of exposure time. In contrast, dissolution measurements using AAS show < 5% dissolution after the same exposure time.^[^ ^49^ ^]^​

Conversely, quantitative measurement requires an additional separation step of particulates from dissolved species, and dissolution measurement is highly reliant on the detection technique. Once the effective separation is ensured, a range of direct/indirect analytical techniques can be used for measurements (e.g., ICP‐MS, AAS). Each of the mentioned methods has its own sensitivity and detection limits. Notably, all dissolution assessment methods work primarily on particulate to bulk level and fail to capture aspects related to complexation, speciation and nucleation, and morphological changes induced on the particles. In addition to separation methods and detection techniques, slight changes in the experimental parameters (e.g., particle concentrations) affect the dissolution kinetics. For instance, Keller et al (2021) explore how dissolution kinetics vary among different nanoforms (NFs) of materials under simulated lung fluid conditions using a continuous flow system and ICP‐MS. By testing 17 NFs, including silica, metal oxides, and pigments, the study found that dissolution rates and half‐times spanned several orders of magnitude, influenced by NF size, shape, surface area, and surface treatment. ICP‐MS precisely quantified dissolved ions, revealing significant differences in dissolution kinetics across concentrations and environments.^[^ ^58^ ^]^

The continuous flow method has emerged as an effective tool for assessing ENM dissolution kinetics, offering accurate, real‐time data across a wide range of NFs. It excels in simulating dynamic, realistic exposure conditions, preventing ion saturation, and enabling the capture of dissolution half‐times that span from hours to years. This approach has been particularly useful for highly soluble ENM, especially in biological media, where dissolution behavior closely aligns with the in vivo environment.^[^ ^59^ ^]^ The integration of ICP‐MS further enhances the method's sensitivity, allowing precise quantification of dissolved ions and providing valuable insights into dissolution processes. However, the method is not without limitations. It requires large material quantities, which can be a constraint for rare or expensive ENM, and involves technically complex setups that demand significant resources for maintenance. Its application in low‐solubility materials, such as TiO_2_, SiO_2_, and Fe_3_O_4_, and complex environmental matrices remains challenging due to issues like high agglomeration rates, complexation, and re‐precipitation of dissolved metals.^[^ ^58^ ^]^ These challenges are further compounded by difficulties in detecting long‐term dissolution behaviors and the need to optimise test concentrations, durations, and media flow rates. Moreover, simulated environmental conditions such as ASW or river water, characterised by low dissolving environments, often fail to detect dissolved species at the initial stages of experiments. Despite these drawbacks, the continuous flow method remains a robust and valuable approach for studying ENM dissolution under controlled yet dynamic conditions. It provides critical data that supports the development of safer and more sustainable ENM, highlighting its potential as a cornerstone in advancing dissolution research and SbD initiatives.

The need to develop new techniques for *in situ* measurement of ENM dissolution is essential to reduce redundancy in data and minimise the probability of over‐/underestimation of measurement of dissolved species. Proton Nuclear Magnetic Resonance (^1^H NMR) is a powerful tool that provides real‐time, in‐line monitoring of dissolution kinetics with high sensitivity, even for low concentrations.^[^ ^60^ ^]^ Using an NMR flow cell, the method measures changes in the chemical environment of protons, directly reflecting the concentration of dissolved species. Unlike UV spectroscopy, which can suffer from signal overlap, ^1^H NMR offers high selectivity, enabling precise quantification of dissolution rates in multi‐component systems. Studies have demonstrated strong correlations between CuO nanoparticle dissolution patterns measured using ^1^H NMR spin‐spin relaxation times and traditional techniques like GF‐AAS and dialysis, highlighting its reliability across various media.^[^ ^61^, ^62^ ^]^ Despite its advantages, monitoring NM dissolution remains technically challenging due to the dynamic nature of the process. Nevertheless, technological innovations continue to enhance our understanding of NM properties, structures, and dissolution behavior.

The use of stable isotope enrichment (Figure 3B) on the ENM proved to be a reliable and powerful alternative toward accurate detection and precise quantification of ENM dissolution, accumulation, and biodistribution, at a concentration as low as environmentally realistic concentration.^[^ ^63^, ^64^ ^]^ The significance of this innovative approach was realised when Yu et al. (2017) used the double isotope labeling method to investigate the intracellular dissolution of Ag NPs. The authors used two stable isotopes of Ag (^107^Ag^+^ and ^109^Ag^+^) in their studies.^[^ ^65^ ^]^ They concluded the changes in intracellular isotope ratio of Ag with time on exposure to the isotope‐labeled Ag NPs. This was another breakthrough study that allowed the use of stable isotope labeling as a strategy to assess dissolution in a complex *milieu*. Similarly, the paper by Yang et al. (2018) highlights the application of dual stable isotope labeling to study the uptake, transformation, and translocation of Ag NPs and silver ions (Ag⁺) in rice plants. Using isotopes ¹⁰⁷Ag and ¹⁰⁹Ag, the authors effectively tracked the transformation of Ag NPs into Ag⁺ through oxidation and vice versa via reduction. This approach demonstrated that Ag NPs primarily accumulated in roots, while Ag⁺ was more prevalent in shoots, indicating in vivo oxidation of Ag NPs during translocation. The study underscores the advantages of stable isotope labeling, such as precise tracking of distinct forms of silver in complex biological systems, while providing critical insights into ENMs behavior and potential ecological implications.^[^ ^66^ ^]^

**Figure 4:**
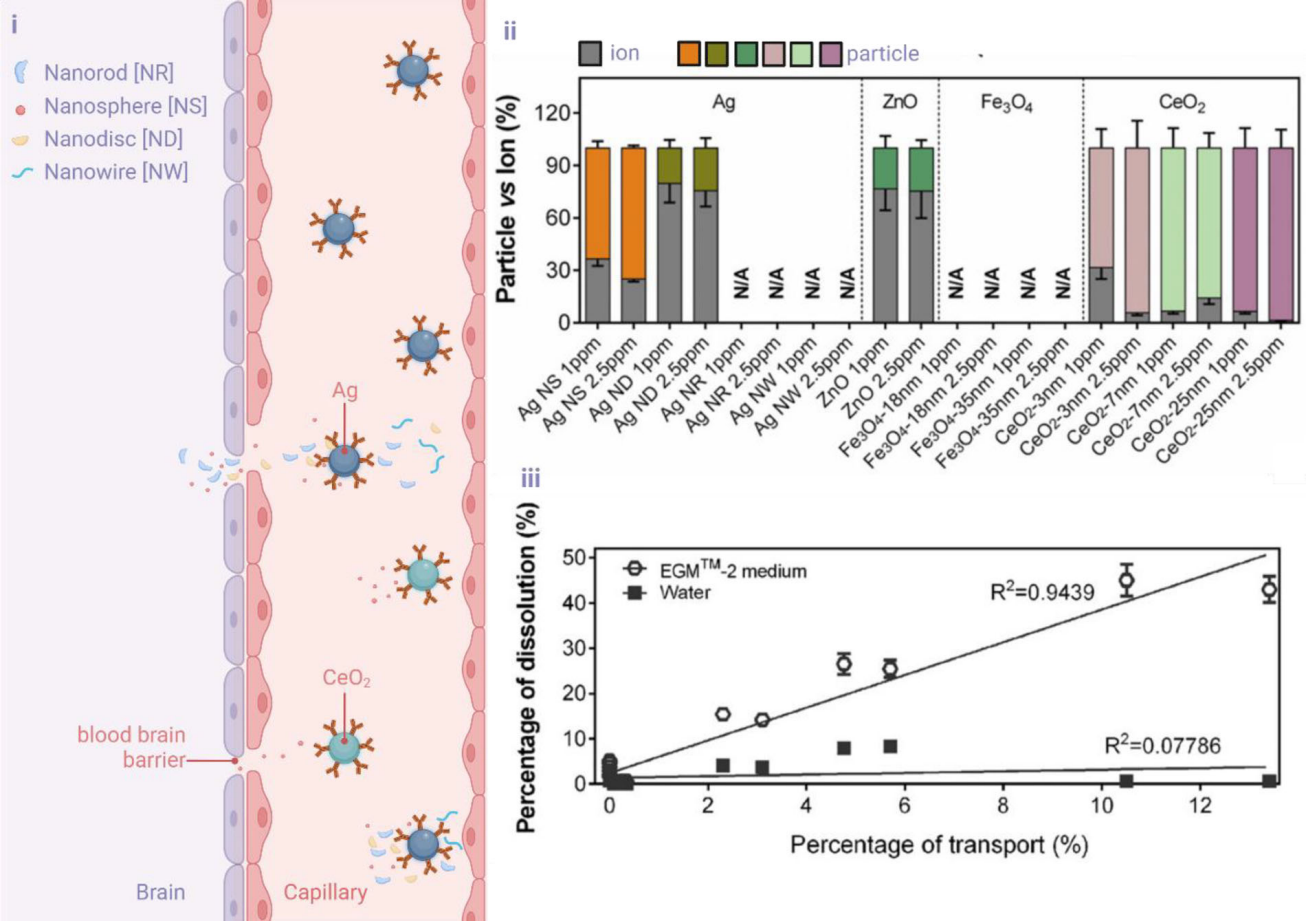
Evaluation of dissolution and transport behavior of nanomaterials using sp‐ICP‐MS. (i) Schematic representation of nanomaterials (nanorods [NR], nanospheres [NS], nanodiscs [ND], and nanowires [NW]) crossing the blood‐brain barrier, with ionic and particulate forms depicted. (ii) Quantification of the relative proportions of particle versus ion forms for Ag, ZnO, Fe₃O₄, and CeO₂ nanoparticles across different concentrations and sizes. Data illustrate dissolution behavior, with higher ion release observed for Ag nanoparticles compared to other materials. (iii) Correlation between percentage dissolution and percentage transport of nanomaterials across barriers in different media (EGM™‐2 and water). Results demonstrate a strong correlation in EGM™‐2 medium (*R* ^2^ = 0.9439), emphasising the influence of media composition on dissolution and transport kinetics. This figure highlights the application ofsp‐ ICP‐MS for analysing the dissolution, transport, and transformation of ENM in biologically relevant systems. Figures were adapted with copyright permission from^[^ ^29^ ^]^ (Figure made using Biorender software).

Another method that revolutionised the *in situ* measurement of ENM dissolution is the use of sp‐ICP‐MS, wherein the ENMs present in the aqueous medium are purged into the plasma through a nebuliser similar to that of the digested/dissolved samples, but using high acquisition rates. The method provides flexibility to analyse the samples both in dissolved ions and particle mode, depending on the user's needs. Researchers have used this technique to investigate the transformation of a range of ENM (ZnO, CeO_2_, Fe_3_O_4_, Ag) in several complex media viz. biological media^[^ ^29^ ^]^ municipal wastewater.^[^ ^67^ ^]^ Notably, Guo et al (2021) used this technique to investigate the transformation of a range of ENM (ZnO, CeO_2_, Fe_3_O_4_, Ag) in the cell culture media​.^[^ ^29^ ^]^​ The technique enabled a dynamic understanding of ENM present in the form of particulates and dissolved species. However, measuring the dissolution of ENMs for highly agglomerating samples (such as Fe_3_O_4_) remains challenging. **Figure** **4** provides a schematic representation of the dissolution and transport behavior of ENM using sp‐ICPMS, showing higher ion release for Ag nanoparticles compared to ZnO, Fe₃O₄, and CeO₂ across various sizes and concentrations. A correlation between dissolution and transport was observed in the EGM‐2 medium (*R* ^2^ = 0.9439), highlighting the role of media composition in influencing ENM dissolution and transformation. Other studies utilised sp‐ICP‐MS to precisely quantify the dissolution of Ag NPs in complex environmental matrices. In the study by Azodi et al. (2016), sp‐ICP‐MS was employed to track the dissolution of polyvinylpyrrolidone (PVP)‐coated Ag NPs (80 nm) in municipal wastewater and deionised water, showing dissolved silver concentrations of 0.89 ± 0.05 ppb after 168 h in wastewater.^[^ ^67^ ^]^ Challenges included lower dissolution rates in wastewater due to sulfide interactions and secondary nanoparticle formation (≈22 nm), highlighting matrix effects on dissolution quantification. Similarly, Mitrano et al. (2014) studied 60 nm and 100 nm Ag NPs under different water chemistries, revealing significant size reductions in deionised water within 24 h but slower dissolution in chloride‐ and sulfide‐rich environments.^[^ ^68^ ^]^ While sp‐ICP‐MS effectively measured dissolution down to environmentally relevant concentrations, limitations included the under‐detection of nanoparticles below 10 nm and signal interference from complex matrices. To reduce the complexities in analysing dissolution using sp‐ICP‐MS, there is a need for further optimissation in method development for relatively unstable ENM. Next‐generation ICP‐MS technologies and multi‐collector ICP‐MS instruments can further drive accuracy in both particle and ion measurements and a more reliable understanding of the dissolution process.

**Figure 5:**
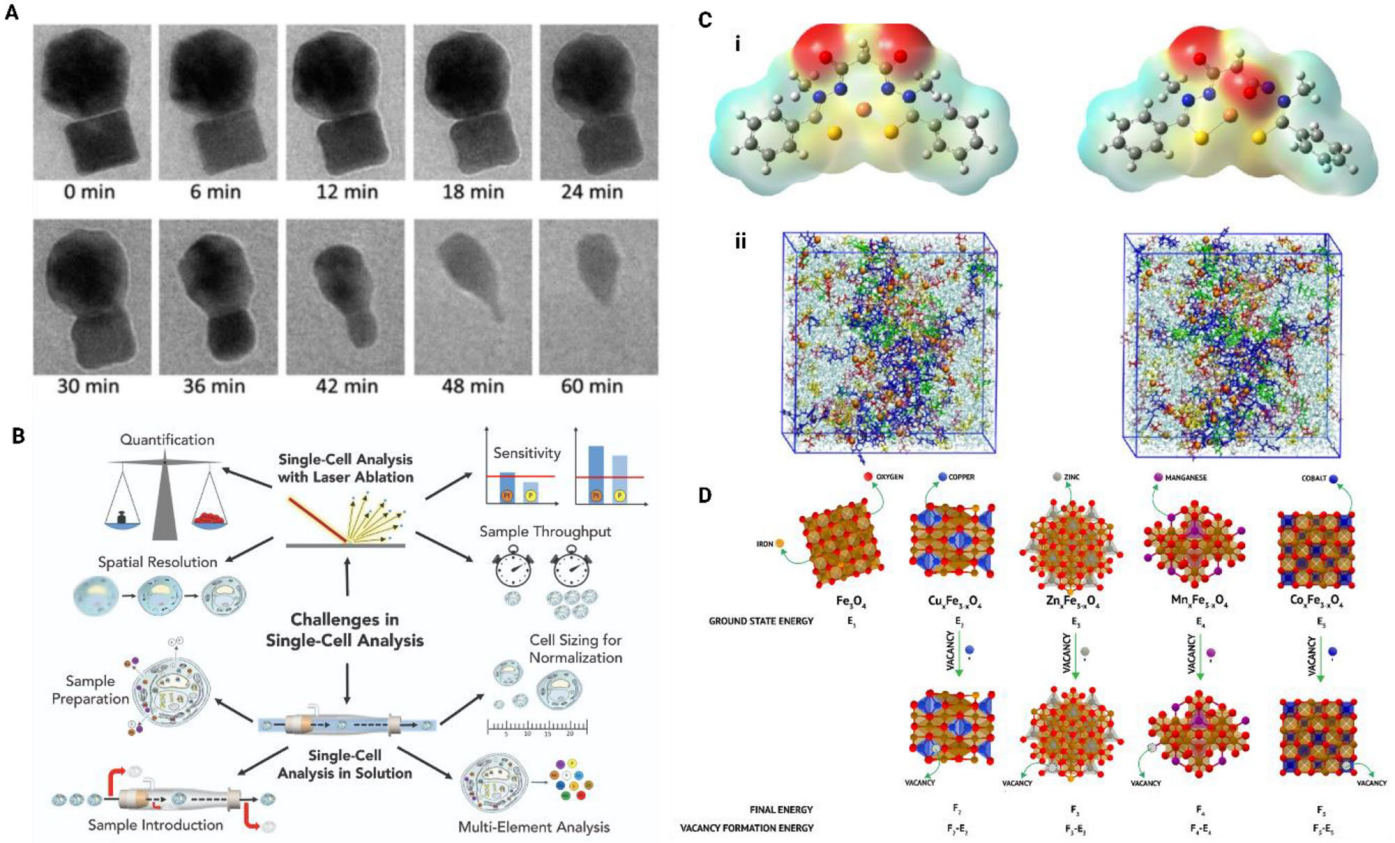
Multidimensional analysis of nanomaterial behavior using advanced techniques. (A) Time‐resolved TEM images illustrate the dissolution process of ENM at the nanoscale, highlighting the progressive morphological changes over 60 min.^[^ ^28^ ^]^ (B) Schematic representation of challenges in single‐cell analysis using laser ablation coupled with ICP‐MS. Key aspects include spatial resolution, sensitivity, sample throughput, and sample preparation, enabling multi‐elemental quantification at the cellular level. (C) (i) Molecular structures showing surface interactions of nanomaterials with reactive species. (ii) Molecular dynamics simulations of nanomaterial interactions in solution, capturing dissolution and reconfiguration behaviors at the atomic level.^[^ ^75^ ^]^ (D) Structural insights into Fe₃O₄ and doped Fe₃O₄ variants (Cu, Zn, Mn, Co) highlighting vacancy formation energies and corresponding atomic configurations. These illustrate the impact of dopants on the stability and functionality of the nanomaterials.^[^ ^24^ ^]^ This figure integrates experimental and computational approaches to provide a comprehensive understanding of nanomaterial dissolution, transport, and structural modifications. (Figure made using Biorender software).

Another newly established operation mode for ICP‐MS, described as single‐cell inductively coupled plasma mass spectrometry (sc‐ICP‐MS) has been employed to assess the intracellular dissolution of ENM by quantifying metal content within individual cells. For instance, Suárez‐Oubiña et al. (2023) utilised sc‐ICP‐MS to evaluate titania (TiO₂) and Ag NPs associations in cell lines from aquaculture species, determining that citrate‐coated TiO₂ nanoparticles primarily interacted with cell membranes, while polyvinylpyrrolidone‐coated Ag nanoparticles were internalised by the cells.^[^ ^69^ ^]^ Similarly, Gimenez‐Ingalaturre et al. (2024) applied sc‐ICP‐MS to study silver interactions with bacteria, revealing that bacteria exposed to Ag NPs accumulated less silver compared to those exposed to ionic silver, with the latter being fully internalied, indicating higher bioavailability of ionic silver. While sc‐ICP‐MS offers precise quantification of metal content in single cells, challenges remain in distinguishing between internalised nanoparticles and those adhering to cell membranes. Additionally, the technique may face limitations (**Figure** **5**B) in detecting ENM below certain size thresholds and in complex biological matrices, potentially affecting the accuracy of dissolution assessments.

Synchrotron radiation techniques, including hard X‐ray nanoprobe and X‐ray absorption near‐edge structure (XANES) imaging, have been instrumental in assessing the intracellular and intra‐organism dissolution of ENM. These methods enable high‐resolution, element‐specific imaging and chemical state analysis within biological specimens. For instance, Pattammattel et al. (2020) employed hard X‐ray nano‐XANES to achieve nanoscale chemical imaging with high sensitivity, facilitating the study of chemical states of nanoscale materials.^[^ ^70^ ^]^ Additionally, a review by Borfecchia et al. (2018) discusses the capabilities of X‐ray microbeam and nanobeam techniques, highlighting their applications in materials characterisation, which can be extended to studying nanoparticle interactions within biological systems.^[^ ^71^ ^]^ Hard X‐ray Nanoprobe and XANES imaging were effectively employed to study the dissolution and transformation of ZnO nanomaterials under real‐world conditions, such as municipal wastewater and primary sludge. These high‐resolution methods enabled spatially resolved chemical mapping at the nanoscale, providing critical insights into how ZnO transformed into secondary phases like ZnS and Zn–phosphate. XANES imaging revealed rapid ZnO transformation, with ZnS emerging within an hour and Zn–phosphate becoming dominant after three hours, influenced by sludge chemistry. The integration of these tools offered clarity in studying nanomaterial transformations in complex matrices, overcoming limitations of bulk analytical techniques, and advancing environmental impact assessments.^[^ ^72^ ^]^ These studies highlight the efficacy of synchrotron‐based hard X‐ray nanoprobe and XANES imaging in elucidating the intracellular behavior and dissolution of nanoparticles, providing critical insights into their biotransformation and potential impacts on organismal health.

Recent years have seen a rise in experimental techniques capable of assessing ENM dissolution with higher levels of confidence. Proton nuclear magnetic resonance (NMR) has been used to characterise reactivity,^[^ ^61^ ^]^ while stable isotope enrichment allows for detecting dissolution at trace levels.^[^ ^73^ ^]^ sp‐ICP‐MS has enabled real‐time measurement of both particulate and dissolved ions, enhancing dissolution characterisation in biologically relevant settings.^[^ ^29^ ^]^ In the past few years, the integration of computational models with these methods has yielded significant progress in understanding dissolution at scales not achievable experimentally, providing insights into SbD potential. Due to collaborative efforts in experimental assessment and computational modeling, our understanding of NM dissolution now offers deeper insights into mechanisms and kinetics. Predictive models not only mimic experimental dissolution trends but also allow for robust safety screening, providing a basis for regulatory applications and reducing testing burdens in future SbD efforts. Moving forward, adopting dissolution as a regulatory metric could enhance SbD's role in achieving safer and more sustainable, industrial‐scale processes and, ultimately, eco‐friendly innovations. Lastly, advanced techniques such as *in situ *liquid cell TEM allows to study the NP‐exposure media interaction from a sub‐nanometer level to a particulate level. Recent advances in TEM holder technology have allowed researchers to perform growth and nucleation‐related experiments in situ. Particles having surface features with a smaller radius of positive curvature (convex) tend to be energetically unstable and can thus have preferential dissolution and higher equilibrium solubility.^[^ ^4^ ^]^ Though ENMs are often described as spherical, they are highly faceted, with different surface tensions in different surface planes. Liu et al. (2008) showed that for galena (PbS) NPs, {1 1 1} and {1 1 0} faces with lower coordination numbers dissolve faster than {1 0 0} faces of nanocrystals.^[^ ^74^ ^]^ Wu et al. (2017) through liquid cell TEM (Figure 5A) showed that the oxidative etching of NPs largely depends on the atomic location on the surface as a factor of time.^[^ ^28^ ^]^ They also developed quantitative dissolution kinetic models by plotting mass loss versus time in the electrolytic solution. **Table** **1** highlights the major advancements and limitations of various dissolution assessment techniques.

**Table 1:** Comparison of Key Experimental Methods for Assessing ENM Dissolution Dynamics.

Method	Measurement Principle	Strengths	Limitations	Typical Applications	Representative Refs.
Static Batch (e.g., Filtration, Dialysis)	ENM are incubated in a controlled medium for a set period; dissolved ions are separated from nanoparticles by filtration/dialysis/centrifugation/ combination of them	Straightforward, low‐cost setup; Good for equilibrium solubility tests; Accessible to most labs.	May not capture real‐time kinetics; Potential over‐/under‐estimation if diffusion or separation is incomplete; Dialysis can lag actual dissolution, especially for fast‐dissolving ENM. Limited by the MWCO of the membranes	Preliminary hazard/risk assessments; Comparative screening of multiple ENMs or media; Studying equilibrium solubility.	[18, 42, 51]
Continuous Flow (Dynamic Dissolution)	ENM are continuously exposed to a flowing medium (e.g., FFF); effluent analysed at intervals (e.g., by ICP‐MS, AAS) to track ion release.	Real‐time dissolution kinetics; Prevents ion saturation; Closer to physiological or environmental flow conditions.	Requires large sample quantities; Complex, often expensive setups; High agglomeration or re‐precipitation may interfere.	Simulating bloodstream or river flow; Capturing rapid dissolution phases; Studying dissolution over short or long timescales.	[47, 54, 55]
Dialysis	Nanoparticles and ions are separated by a semipermeable membrane; ions diffuse based on concentration gradients.	Simple experimental design; Small sample volumes; Low cost.	Can underestimate or delay dissolution kinetics due to slow diffusion; Membrane interactions and fouling may bias results. Limited by the MWCO of the membranes	Preliminary dissolution studies; Screening different ENM types; Low‐throughput or teaching labs.	[19, 22]
Centrifugal Ultrafiltration	A centrifugal force drives dissolved ions through an ultrafiltration membrane, retaining nanoparticles above a certain size threshold.	Faster separation than dialysis; Efficient at retaining ENM while collecting dissolved species; Recommended in OECD guidelines.	Requires multiple centrifugation steps; Membrane selection crucial to avoid clogging or unspecific binding; Risk of particle aggregation or sedimentation affecting separation.	Generating dissolution profiles in complex media; Confirming extent of ion release; Regulatory compliance testing.	[22, 56]
Single‐particle ICP‐MS (sp‐ICP‐MS)	Individual nanoparticles are ionized in a plasma, producing time‐resolved signals to distinguish dissolved ions from particles.	Real‐time detection of particles and ions; High sensitivity at low concentrations; Differentiates particulate vs ionic forms.	Limited size resolution (typically ≥10 nm); Matrix effects can distort results; Highly agglomerating ENM pose analytical challenges.	Environmental fate in complex matrices; Biological exposure studies; Tracking transformations in real‐time.	[67, 68]
Single‐Cell ICP‐MS (sc‐ICP‐MS)	Quantifies metal content in individual cells; helps track intracellular dissolution or uptake of ENM at cellular resolution.	Resolves cell‐to‐cell variations; Useful for intracellular dissolution and biodistribution; Complements in vitro toxicity assays.	Distinguishing surface‐bound vs internalized ENM is difficult; Limited sensitivity for very small ENM; Complex sample prep can introduce artifacts.	Intracellular fate and speciation; Toxicity and mechanistic studies; Biouptake assessments.	[69, 75, 76]
Stable Isotope Labeling	ENM isotopically labeled (e.g., ^107^Ag, ¹⁰⁹Ag) to distinguish dissolution products from background or unlabeled species.	Ultra‐trace detection; Allows precise tracking of ions vs particles; Minimal interference from matrix elements.	Synthesis of labeled ENM can be expensive or complex; Requires specialized ICP‐MS capabilities; Some isotopes are not readily available.	Environmental fate in complex or low‐concentration systems; Intracellular or plant uptake studies; Ecosystem‐level tracking.	[63, 65, 66, 73]
Liquid‐Cell TEM	Direct imaging of nanoparticle dissolution in the liquid environment at near‐atomic resolution, capturing morphological changes in real‐time.	Visualizes dissolution front, growth sites, morphological evolution; Ultra‐high spatial resolution.	Requires specialized TEM holders and vacuum compatibility; Limited sample size/volume; Beam damage can alter dissolution behavior.	Fundamental mechanistic studies; Observing facet‐specific dissolution; Real‐time morphological changes.	[^28^]
NMR (^1^H NMR Flow Cell)	Measures change in proton environment and relaxation times to infer the concentration of dissolved species in real‐time.	Real‐time, in‐line measurement; High sensitivity even at low concentrations; No need for sample destruction or elaborate sample prep.	Equipment and setup can be costly; Require careful calibration for multi‐component systems; Typically less routine for high‐throughput dissolution assessments.	Continuous monitoring of dissolution kinetics; Multiphasic or multi‐component media; Linking dissolution data to reaction byproducts.	[61, 62]
Synchrotron‐Based Techniques (e.g., XANES, Hard X‐ray Nanoprobe)	High‐energy X‐rays provide element‐specific imaging and speciation; can map oxidation states and transformations at nanoscale resolution.	Unrivaled sensitivity and spatial resolution; Chemical state and local coordination insights; Applicable in situ for complex samples.	Limited facility availability and beamtime; Complex sample prep for hydrated specimens; Data interpretation can be challenging.	Mechanistic transformation studies; Tracking formation of secondary phases; High‐resolution imaging of chemical speciation in situ.	[70, 71]
Computational‐Experimental Integration (e.g., MD + spICP‐MS)	Hybrid approach coupling experimental data with simulations (MD, DFT) to refine dissolution mechanism predictions and track real‐time dissolution.	Predictive power with reduced experimentation; Identifies mechanistic details difficult to resolve in experiments alone; High‐throughput screening.	Requires robust computational resources/expertise; Validation depends on reliable experimental inputs; Some complexities difficult to capture accurately.	Advanced SbD framework; Virtual labs for accelerated materials testing; Direct linking of molecular‐scale insights to macroscale dissolution.	[77, 78]

## 4 Implication of ENM Dissolution in Safety‐by‐Design (SbD)

Dissolution plays a crucial role in the SbD of ENM due to its significant influence on biochemical speciation and the complete transformation of materials. Understanding how ENM dissolve and transform in biological or environmental media is critical for predicting their behavior in real‐world applications.^[^ ^23^ ^]^ For example, the dissolved ions from ZnO nanoparticles can perturb the structure of enzymes such as acetylcholine esterase, affecting neurotransmission and potentially leading to neurotoxicity.^[^ ^79^ ^]^ These interactions highlight the importance of considering the material's physical as well as chemical transformations when designing nanoparticles for safety.

**Figure 6:**
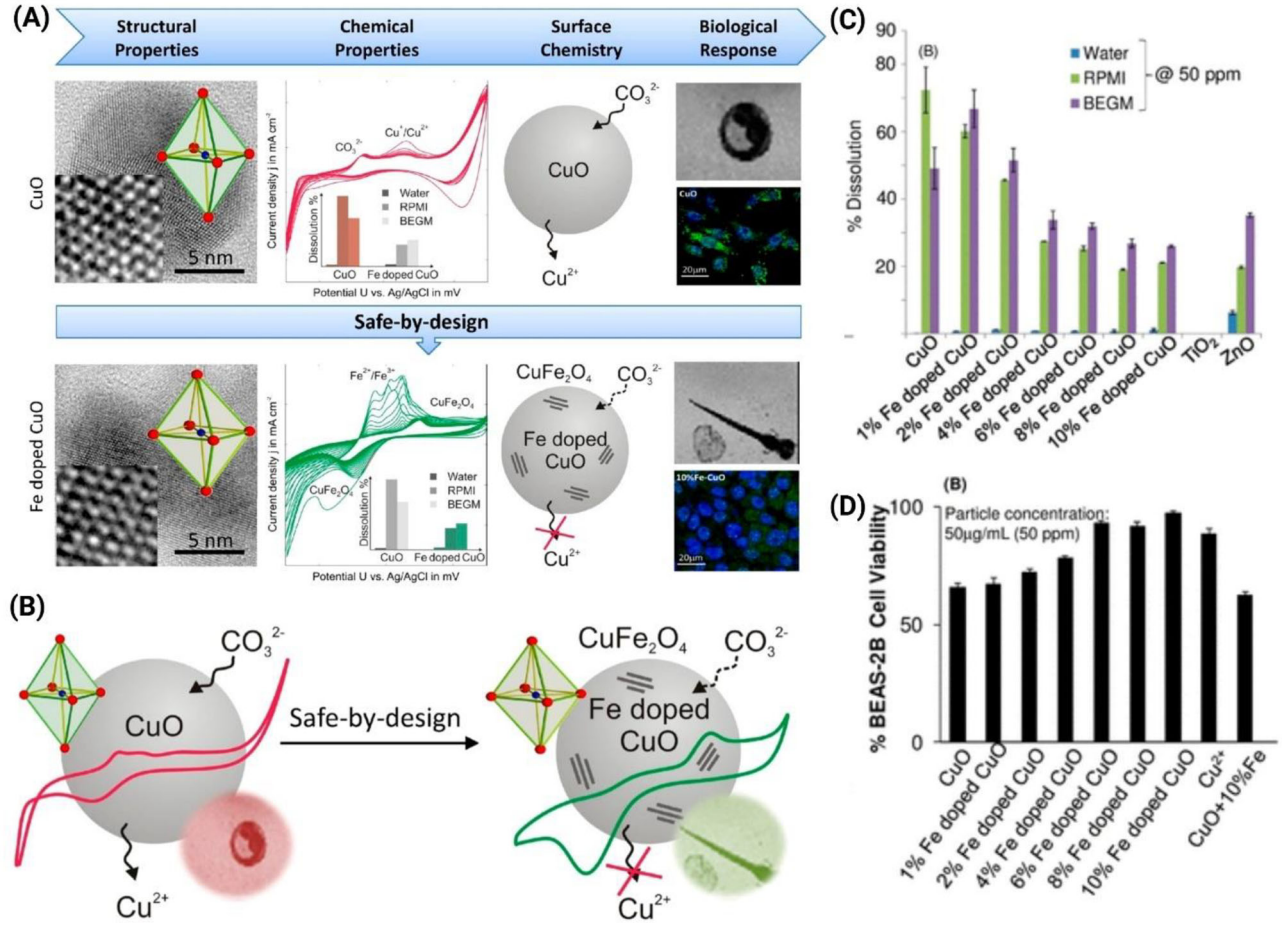
SbD approach for modulating the dissolution behavior and biological response of CuO nanoparticles through Fe doping. (A) Structural, chemical, surface, and biological analysis of CuO and Fe‐doped CuO nanoparticles. High‐resolution TEM images illustrate the atomic structure of CuO and Fe‐doped CuO. The electrochemical profiles highlight the altered redox behavior in Fe‐doped CuO, with a reduction in Cu^2^⁺ ion release across different media (water, RPMI, and BEGM). Surface modifications and the formation of CuFe₂O₄ phases upon Fe doping reduce dissolution and toxicity. Fluorescent microscopy images show reduced cellular uptake of Fe‐doped CuO compared to CuO. (B) Schematic representation of the SbD strategy to reduce Cu^2^⁺ release and improve nanoparticle safety by introducing Fe doping. (C) Percentage dissolution of CuO and Fe‐doped CuO nanoparticles (1% to 10% Fe doping) across various media, highlighting reduced dissolution with increasing Fe content. (D) BEAS‐2B cell viability analysis shows improved biocompatibility of Fe‐doped CuO compared to pristine CuO, demonstrating the potential of Fe doping to mitigate cytotoxic effects. This figure showcases the effectiveness of Fe doping in aligning nanomaterial dissolution with SSbD framework to reduce environmental and biological risks. All figures were reproduced with copyright permission from^[^ ^25^ ^]^ (Figure made using Biorender software).

SbD strategies often employ techniques such as doping to modulate the dissolution behaviors of nanoparticles without altering their intrinsic properties. For example, doping CuO nanoparticles with iron has been shown to reduce their dissolution rate, thereby decreasing their toxicity. Fe as a dopant in CuO nanostructures reduces the extent of dissolution without affecting the intrinsic properties of CuO nanoparticles. Consequently, there was a significant reduction in the toxicity of CuO nanoparticles with an increase in the dopant concentration. However, the strategy is not universal to all ENM. The correlation between ENM transformation, stability, uptake, and cytotoxicity is pivotal to making conclusive arguments on the SbD of ENMs. **Figure** **6** illustrates the SbD strategy of Fe doping in CuO nanoparticles, which significantly reduces Cu^2^⁺ ion release and dissolution across various media, as well as cytotoxicity, compared to pristine CuO. The introduction of Fe forms stable CuFe₂O₄ phases, improving biocompatibility and aligning nanomaterial design with SbD framework to mitigate environmental and biological risks. In addition, the interplay between most of these observations will vary on a case‐by‐case basis of ENM variation, making it challenging to establish general guidelines. The extent and the rate of dissolution certainly would vary with the introduction of dopants, but doping as a strategy toward the vision of SbD is still being investigated. Furthermore, the environmental and biological media in which ENM are deployed play a critical role in their dissolution and subsequent transformations. The presence of certain ions or molecules can either accelerate or inhibit dissolution. For instance, chloride ions in a cellular medium can form a passivating layer on the surface of nanoparticles, slowing their dissolution. Ag NPs under different water chemistries, reveal slower dissolution in chloride‐ and sulfide‐rich environments.^[^ ^68^ ^]^ The concept of dissolution‐driven degradability presents a novel SbD strategy, aimed at developing ENM that not only fulfill their intended function but also break down into environmentally benign byproducts after use. This approach is particularly important in fields such as medicine, agriculture, and consumer products, where the lifecycle impact of materials is critical. For instance, in medicine, biodegradable nanoparticles can be designed to deliver drugs effectively and then dissolve harmlessly within the body, reducing the risk of long‐term toxicity. A practical example is the development of polymeric nanoparticles for cancer therapy, where the particles are designed to release their medicinal payload at the target site and then degrade into non‐toxic substances that are easily excreted by the body.^[^ ^80^ ^]^

In agriculture, ENMs engineered to degrade under specific environmental conditions can deliver nutrients or pesticides to plants efficiently. Once their function is completed, these particles degrade, minimising the accumulation of harmful residues in the ecosystem.^[^ ^81^ ^]^ For instance, Carmona et al (2020) studied calcium phosphate (CaP) nanoparticles in detail for their dissolution kinetics and nutrient release, influenced by morphology. Spherical nanoparticles release Ca^2+^ ions faster than nanoplatelets, which exhibit a gradual release. Amorphous CaP nanoparticles show rapid nitrate loss, while nanoplatelets, with slower nitrate release, suggest controlled dissolution useful in agriculture. This tailoring of ENMs design enhances the efficiency and sustainability of nanofertilissers by optimiing nutrient delivery in soil. Such insights are crucial for developing nanofertilisers that reduce environmental impact and improve agricultural productivity.^[^ ^82^ ^]^

In real‐world applications, ENM are commonly integrated into diverse organic or inorganic matrices—ranging from polymers in consumer products to composite coatings in construction materials. Such matrices can substantially affect dissolution by physically shielding ENMs from their surrounding environment, altering local pH or ionic strength, or triggering secondary reactions at the interface. For instance, polymeric coatings have been shown to retard ion release by limiting direct particle contact with aqueous media, whereas mineral substrates may sequester dissolved species through precipitation, effectively modifying both dissolution kinetics and overall environmental fate.^[^ ^83^ ^]^ Accounting for these substrate–ENM interactions is crucial when extrapolating laboratory data to practical scenarios, as matrix effects can significantly diverge from dissolution behaviors observed in free‐particle studies, ultimately informing more accurate safety evaluations and underpinning robust SbD approaches. Similarly, in consumer goods, biodegradable nanocomposites can enhance product functionality while ensuring that post‐consumer waste degrades into non‐toxic components. This approach is critical in packaging applications where the environmental impact of waste materials is a growing concern. A notable case is the use of nanocellulose in packaging materials, which offers enhanced barrier properties and mechanical strength, yet remains fully biodegradable, contrasting sharply with traditional plastic packaging.^[^ ^84^ ^]^ By integrating dissolution‐driven degradability into the design of ENM, manufacturers can align product innovation with environmental stewardship, paving the way for a new era of materials that combine performance with sustainability and safety. This strategy not only mitigates the ecological risks associated with nanomaterials but also aligns with global sustainability goals, making it a quintessential component of future material science developments.

It is important to note that reducing dissolution is only one aspect of a comprehensive SbD strategy. Beyond modulating dissolution rates, SbD encompasses substituting toxic constituents with safer or benign materials, utilising protective shells or coatings to limit ion release, and exploring core–shell architectures that further minimise hazardous interactions. For instance, designing intrinsically non‐toxic cores or replacing high‐toxicity dopants with biocompatible elements can greatly diminish overall risk while maintaining the desired functionality. By combining these complementary strategies, researchers and manufacturers can broaden the scope of SbD to achieve safer and more sustainable nanomaterial applications across various sectors.

Ensuring the safety of ENM systematically through the SbD approach involves a structured and iterative evaluation across their entire lifecycle, particularly during design modifications such as doping, surface functionalisation, and composite formation. Systematic safety evaluation typically employs tiered approaches, beginning with predictive computational modeling and in vitro assays to rapidly screen for potential hazards (e.g., cytotoxicity, oxidative stress induction, and dissolution kinetics). Subsequently, selected candidate materials undergo more comprehensive and realistic assessments, including advanced in vitro (e.g., ssc‐ICP‐MS, organ‐on‐chip systems) and *in situ* techniques (e.g., synchrotron‐based XANES and XRF imaging), to precisely characterize dissolution behavior, bioavailability, and transformation dynamics under physiologically relevant conditions.^[^ ^29^, ^73^, ^79^ ^]^ During modifications such as doping or composite synthesis, safety assessments must systematically evaluate changes in material stability, secondary product formation, and interactions with biological and environmental media, ensuring that any alterations enhance, rather than compromise, safety. Moreover, thorough characterisation of newly formed surfaces or interfaces in composite materials is crucial, as these sites often exhibit distinct reactivity profiles that differ from individual components.^[^ ^24^, ^25^, ^85^ ^]^ Establishing robust criteria for material safety—including toxicity thresholds, dissolution rates, and biocompatibility metrics—and integrating these criteria into iterative feedback loops for material refinement enables researchers and industry stakeholders to anticipate and mitigate potential risks proactively. Ultimately, embedding systematic evaluation frameworks within the SbD process facilitates not only safer innovation but also supports regulatory acceptance and sustainability objectives.

## 5 Landscape of Simulation‐Based Models in Understanding Dissolution

Over the past decade, the role of simulation‐based models in understanding ENM dissolution has expanded, evolving into an essential tool for the safe and sustainable development of ENM.^[^ ^34^, ^78^, ^86^ ^]^ Computational techniques such as molecular dynamics (MD), density functional theory (DFT), and, more recently, machine learning (ML) and artificial intelligence (AI), have transformed ENM research by offering a predictive framework that informs the design, lifecycle analysis, and regulatory assessments of ENM.^[^ ^87^ ^]^ This shift toward computational methods has been motivated by the need for efficient, high‐throughput screening techniques to evaluate ENM behavior, reducing the reliance on extensive, costly experimental procedures.

Computational approaches offer insights into the dissolution kinetics and transformation behaviors of ENM in complex media, allowing researchers to anticipate their physicochemical behaviors and biological impacts. For instance, quantitative structure‐activity relationships (QSAR) and quantitative structure‐property relationships (QSPR) models have become popular in dissolution studies. These models use “nanodescriptors,” unique descriptors that represent ENM properties like size, shape, and chemical composition, to predict behavior. By employing data‐driven models like QSAR and QSPR, researchers can virtually screen ENM for properties associated with dissolution and toxicity, enabling early adjustments to improve safety before synthesis.^[^ ^88^ ^]^ A significant example of simulation‐based models in understanding dissolution comes from studies on vacancy formation energy (VFE) using DFT. VFE modeling allows researchers to predict stability and dissolution rates by analyzing energy requirements for vacancy formation within ENM structures. This technique has been particularly useful for doped ferrite nanoparticles, where higher VFE indicates stronger atomic bonds and thus a lower dissolution potential (Figure 5D). These insights help identify stable and bio‐compatible ENM without extensive experimentation, streamlining the design of ENM for SbD applications. Furthermore, simulations can capture atomic‐level interactions with environmental or biological media, revealing binding affinities, charge distributions, and local molecular geometries that are otherwise difficult to study in detail through traditional experimental methods.

Simulation‐based models have evolved from basic predictive tools to sophisticated frameworks that combine data‐driven and physics‐based modeling for high accuracy and relevance. Early approaches, like the Computational Fluid Dynamics (CFD) and Distorted Grid (DG) models introduced by DeLoid et al. (2015), were designed to improve dosimetry in ENM screening. These models simulated particle transport, sedimentation, and dissolution in in vitro systems, enhancing our understanding of dose metrics in complex environments.^[^ ^89^ ^]^ For example, the DG model demonstrated its capability to handle polydisperse ENM suspensions and assess how variables such as particle size and density impact dissolution in biological media. By accurately simulating particle dynamics, these early models provided a foundation for improved NM risk assessments. The integration of advanced techniques like DFT and MD with QSAR and QSPR modeling represents a significant advancement over the last decade. By combining data from experimental studies and simulations, researchers can now generate comprehensive datasets that reflect real‐world conditions. For instance, MD simulations have been employed to study the interactions between dissolved ENM ions and biomolecules within biological environments. In a recent study, MD and DFT were used to explore how dissolved Cu (II) ions from nanoparticles interact with Elesclomol, a chemotherapeutic drug, revealing complex behaviors like competitive binding, charge transfer (Figure 5C), and local molecular geometry changes. Such details are often impossible to capture through empirical methods alone, demonstrating the advantages of simulation models in expanding our understanding of dissolution processes.^[^ ^75^ ^]^

In advancing the computational models for predicting nanomaterial dissolution, the recently introduced Kalapus Equation (KEq) represents a significant breakthrough.^[^ ^90^ ^]^ This new empirical model outperforms traditional logarithmic and pseudo‐first‐order kinetic models by providing higher accuracy in predicting time‐dependent dissolution behaviors of engineered nanomaterials. KEq effectively addresses the limitations of previous models by allowing extrapolation beyond the initial experimental time range, which is critical for long‐term environmental and biological impact assessments. Its ability to predict solubility with minimal error across various conditions not only enhances the accuracy of safety assessments but also aligns with the principles of SbD by anticipating long‐term transformations and toxicity potentials of ENM. This integration will not only enhance the depth of your discussion on computational modeling but also directly tie into the manuscript's focus on incorporating advanced techniques into safety and sustainability frameworks.

Today, simulation‐based models are recognised as vital tools for the predictive assessment of ENM dissolution, and they are increasingly integrated with ML and AI. The combination of physics‐based modeling with ML enables more accurate predictions, as AI algorithms can analyse vast amounts of data to uncover previously unknown relationships between nano descriptors and dissolution behaviors.^[^ ^78^, ^86^, ^87^ ^]^ This data‐driven approach not only enhances the reliability of QSAR and QSPR models but also allows for continuous refinement based on new findings. With ML integration, simulation‐based models are poised to support high‐throughput virtual screenings, facilitating safer and more sustainable material design. Looking forward, there is significant potential to further refine these models to meet regulatory standards. Standardising protocols and nano descriptors for computational dissolution studies could facilitate the regulatory acceptance of simulation data, offering a pathway to streamline safety assessments for new ENM. Future regulatory frameworks could leverage simulation‐based predictions to support the virtual testing of ENM, enhancing transparency and speeding up approvals for safe NM applications. Moreover, the integration of *in situ* experimental techniques like sp‐ICP‐MS and liquid‐cell electron microscopy is expected to play a key role in validating simulation predictions. These advanced tools allow for real‐time dissolution tracking and particle behavior analysis in complex media, providing empirical benchmarks for refining simulation accuracy. The continuous feedback loop created by real‐time validation is expected to bridge gaps between theoretical predictions and practical observations, enhancing model precision and robustness. Beyond environmental and toxicological studies, simulation‐based models are set to expand into fields like biomedicine, agriculture, and electronics, where controlled dissolution is essential. In biomedicine, these models could aid in the design of ENM for targeted drug delivery by predicting dissolution rates and interactions within specific biological conditions (Figure 5C). Similarly, in agriculture, simulations could support the development of ENM for nutrient release in soil, optimising agricultural practices for improved crop yield and soil health. Tailoring simulation models to application‐specific needs will support the safe and sustainable use of ENM across diverse industries.

## 6 Outlook

**Figure 7:**
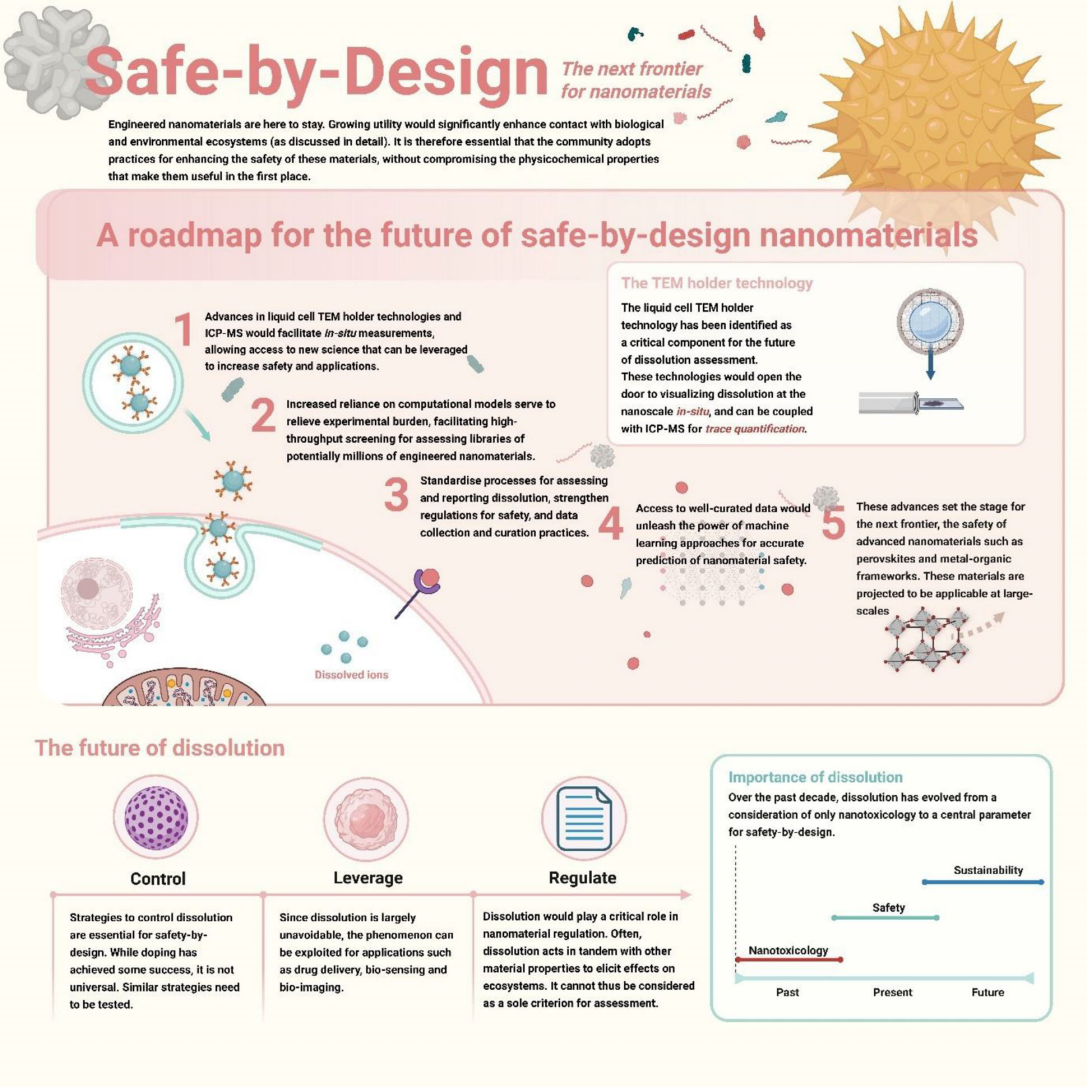
A roadmap for the future of SbD nanomaterials. Advances in analytical techniques such as liquid cell TEM holder technologies and ICP‐MS enable *in situ* measurements of nanomaterial dissolution, fostering increased safety and application potential (Step 1). Computational models further reduce experimental burden, facilitating high‐throughput screening for ENM (Step 2). Standardization of assessment methods strengthens regulatory frameworks and data reliability (Step 3). Access to well‐curated data unlocks machine‐learning approaches for accurate predictions of nanomaterial safety (Step 4). These innovations collectively set the stage for safer, sustainable, and scalable nanomaterials (Step 5). The graphic highlights the importance of dissolution as a critical factor for achieving sustainability, safety, and advanced nanotoxicology in the progression of ENM. (Figure made using Biorender software).

The dissolution of ENM plays a central role in their safety and functionality. SbD framework integrate a deep understanding of dissolution dynamics with innovative engineering strategies to minimize risks while maximising the beneficial applications of nanotechnology. By considering the complex interplay between nanoparticle properties, their dissolution behaviors, and environmental interactions, SbD aims to foster the development of nanomaterials that are not only effective but also inherently safer for both humans and the environment. The advent of advanced analytical techniques such as *in situ* liquid cell TEM, sc/sp‐ICP‐MS, and synchrotron‐based methods (e.g., XANES imaging and hard X‐ray nanoprobe) has transformed our ability to study dissolution processes at unprecedented resolution. These tools enable the exploration of nanoscale transformations, chemical state changes, and spatial distributions within organisms, providing a deeper understanding of dissolution kinetics and associated environmental or biological impacts. For instance, synchrotron techniques offer unparalleled sensitivity for mapping nanoparticle speciation and transformation *in situ*, facilitating their application in complex matrices. Despite these advancements, significant challenges remain. Intracellular dissolution assessments are particularly complex due to the difficulty in separating undissolved nanoparticles from dissolved species within cells. While techniques like sc‐ICP‐MS are promising, they require further optimisation to address issues such as sample preparation, isotopic measurements, and simultaneous multi‐element detection. Similarly, *in situ* synchrotron methods need methodological improvements to enhance their accessibility and applicability to diverse nanomaterials under realistic exposure scenarios. The integration of these experimental approaches with computational models, including DFT and MD simulations, offers an exciting avenue for validating experimental results and accelerating dissolution research. Such models can complement experimental data to provide predictive insights into dissolution kinetics, surface transformations, and interactions with complex media. This synergy opens the door for high‐throughput screening of extensive nanomaterial libraries, reducing research timelines and costs while generating high‐quality datasets for machine learning algorithms. **Figure** **7** presents a roadmap for the future of SbD nanomaterials, emphasising the integration of advanced analytical techniques and computational approaches to enhance safety, sustainability, and scalability. **Step 1** highlights the potential of innovative technologies, such as liquid cell TEM holders and ICP‐MS, to enable *in situ* measurements of nanomaterial dissolution, providing real‐time insights critical for assessing nanoparticle transformations. **Step 2** focuses on leveraging computational models to reduce experimental burden, allowing high‐throughput screening of extensive ENM libraries and enabling predictive insights into their behavior. **Step 3** underscores the importance of standardising dissolution assessment methods to strengthen regulatory frameworks, improve data reliability, and ensure harmonization across studies. **Step 4** explores the power of machine learning algorithms, supported by well‐curated datasets, to generate accurate predictions of nanomaterial safety and environmental impact. Collectively, these advancements pave the way (**Step 5**) for the development of safer and more sustainable ENM, with dissolution emerging as a central parameter in advancing nanotoxicology and sustainability goals. This roadmap highlights the necessity of a multidisciplinary approach to ensure that nanotechnology progresses responsibly while addressing critical environmental and public health challenges.

Looking ahead, the alignment of dissolution insights with SbD framework will be critical for advancing the development of functional yet sustainable nanomaterials. Future efforts should focus on standardizing methodologies, refining experimental techniques, and incorporating synchrotron‐based imaging, computational modeling, and machine learning to address existing gaps. By leveraging these converging strategies, researchers can achieve a deeper understanding of ENM dissolution while promoting safety, sustainability, and innovation across sectors like biomedicine, environmental remediation, and advanced materials design.

## Conflict of Interest

The authors declare no conflict of interest.
